# Impact of Sarcopenia on Spinal Spondylosis: A Literature Review

**DOI:** 10.3390/jcm12165401

**Published:** 2023-08-19

**Authors:** Yuki Kitsuda, Takashi Wada, Shinji Tanishima, Mari Osaki, Hideki Nagashima, Hiroshi Hagino

**Affiliations:** 1 Rehabilitation Division, Tottori University Hospital, 36-1 Nishi-cho, Yonago 683-8504, Tottori, Japan; 2 Division of Orthopedic Surgery, Department of Sensory and Motor Organs, School of Medicine, Faculty of Medicine, Tottori University, Yonago 683-8504, Tottori, Japan; 3 School of Health Science, Faculty of Medicine, Tottori University, 86 Nishi-cho, Yonago 683-8503, Tottori, Japan

**Keywords:** spinal diseases, spondylosis, sarcopenia, muscle mass, literature review

## Abstract

Sarcopenia and spinal spondylosis (SS) are important health challenges among older individuals; however, data regarding the effect of sarcopenia on SS are lacking. Hence, we aimed to organize the existing knowledge on the impact of sarcopenia on SS and explore potential issues in the available literature. We examined the trends and interventions regarding sarcopenia and SS, searching five databases (PubMed, Embase, CINHAL, Web of Science, and Cochrane Library) from inception to January 2023. Sarcopenia-related events were screened, selected, and reviewed, ultimately identifying 19 relevant studies. The identified reports were predominantly retrospective observational studies addressing lumbar degenerative spine disease (LDSD). Sarcopenia could negatively impact the quality of life and postoperative outcomes in several diseases, including cervical spondylotic myelopathy (CSM) and LDSD. However, there was no consensus among the studies regarding the relationship between sarcopenia and pain. These discrepancies were attributed to gaps in the assessment of sarcopenia, which the current study identifies as important challenges. This review identified several problems in the literature, including the limited number of studies examining CSM, adult spinal deformity (ASD) and scoliosis, and the retrospective study design of most reports. The further accumulation of quality research is needed to clarify the relationship between SS and sarcopenia.

## 1. Introduction

Sarcopenia is a progressive and generalized skeletal muscle disorder characterized by an accelerated loss of muscle mass and function. With respect to human health, sarcopenia increases the risk of falls and fractures; impairs the ability to perform activities of day-to-day living; and has been associated with cardiac disease, respiratory disease, and cognitive impairment. Sarcopenia leads to mobility disorders, resulting in a reduced quality of life (QOL), loss of independence or the need for long-term care placement, and death [1]. Therefore, sarcopenia is well-recognized as an important health challenge among the aging population. The prevalence of sarcopenia is higher among individuals with degenerative musculoskeletal diseases (e.g., osteoporosis, osteoarthritis (OA), and spinal spondylosis (SS)) than in the general older population. Sarcopenia is a risk factor for falls and fragility fractures [1], and its prevalence in patients with proximal femoral fractures and vertebral compression fractures is as high as 40%. Moreover, the presence of sarcopenia is a risk factor for postoperative complications and protracted pain after a fragility fracture, worsening the prognosis [2,3,4].

The prevalence of sarcopenia in patients with OA is approximately three times higher than that in the general older population [5]. In addition, the presence of sarcopenia in patients with OA is considered a risk factor for postoperative infections [6] and can hinder postoperative improvement in physical functions [7]. Similar to fragility fractures and OA, SS is also highly prevalent among older individuals, and patients with SS have an increased risk of developing sarcopenia, thereby necessitating appropriate countermeasures; however, reports assessing this issue remain scarce. To date, only lumbar degenerative spine disease (LDSD) has been examined in a meta-analysis, which found that LDSD was associated with a high prevalence of sarcopenia (approximately 25%) and could adversely impact QOL [8]. However, this meta-analysis had several issues, including a small number of included reports, predominantly cross-sectional and retrospective studies, with no high-quality prospective observational or interventional studies. Furthermore, the meta-analysis failed to address cervical spondylotic myelopathy (CSM) or adult spinal deformity (ASD). Accordingly, the association between sarcopenia and SS remains poorly explored. To discuss the need for sarcopenia countermeasures in SS, extensive and comprehensive data are critical to clarify the effects on prognosis and establish interventional strategies.

Therefore, we conducted the present literature review to organize the existing knowledge on the impact of sarcopenia on SS and explore the potential issues in the available literature. 

## 2. Materials and Methods

### 2.1. Database Search

Data were collected in accordance with the procedures recommended in the extension to the PRISMA Statement for Reporting Literature Searches in Systematic Reviews [9]. A comprehensive literature search was performed using five electronic databases, namely, PubMed, Embase, CINAHL, Web of Science, and Cochrane Reviews. For each database, the search range was set from the time of database inception to January 2023. Table 1 presents the keywords used in the search strategy. A manual search was performed using the citations listed in included articles, as needed. 

### 2.2. Study Eligibility

The clinical questions in this review were formulated as follows: patients (P): diagnosed with SS who had been evaluated for sarcopenia; exposure (E): sarcopenia defined according to the European Working Group on Sarcopenia in Older People, the Foundation for the National Institutes of Health, the Asian Working Group for Sarcopenia (AWGS), the Sarcopenia Definitions and Outcomes Consortium, and the International Working Group on Sarcopenia; comparison (C): non-sarcopenia; outcome (O): clinical outcomes (including QOL, physical function, and postoperative clinical outcomes; and study design (S): prospective and retrospective cohorts and interventional studies. To examine the relationship between SS and sarcopenia, only articles that met the above PECOS criteria were selected, and all others were excluded.

### 2.3. Data Collection

Relevant articles were not filtered out; two authors (Y.K. and T.W.) independently selected articles based on the eligibility criteria. Each author checked and screened the abstract and main text of the papers. In cases of disagreement, all the other authors evaluated the paper for eligibility. Two authors (Y.K. and T.W.) abstracted the final articles independently, and key elements were extracted using a predesigned template. The included articles were described with respect to the author information, study design, population (including nationality, disease, and sex), subject details, sarcopenia definition, and assessment methods. The primary endpoints of the studies were recorded as outcomes, and the actual data and results obtained were recorded as the key findings of the results. 

## 3. Results

### 3.1. Search Results

**Figure 1:**
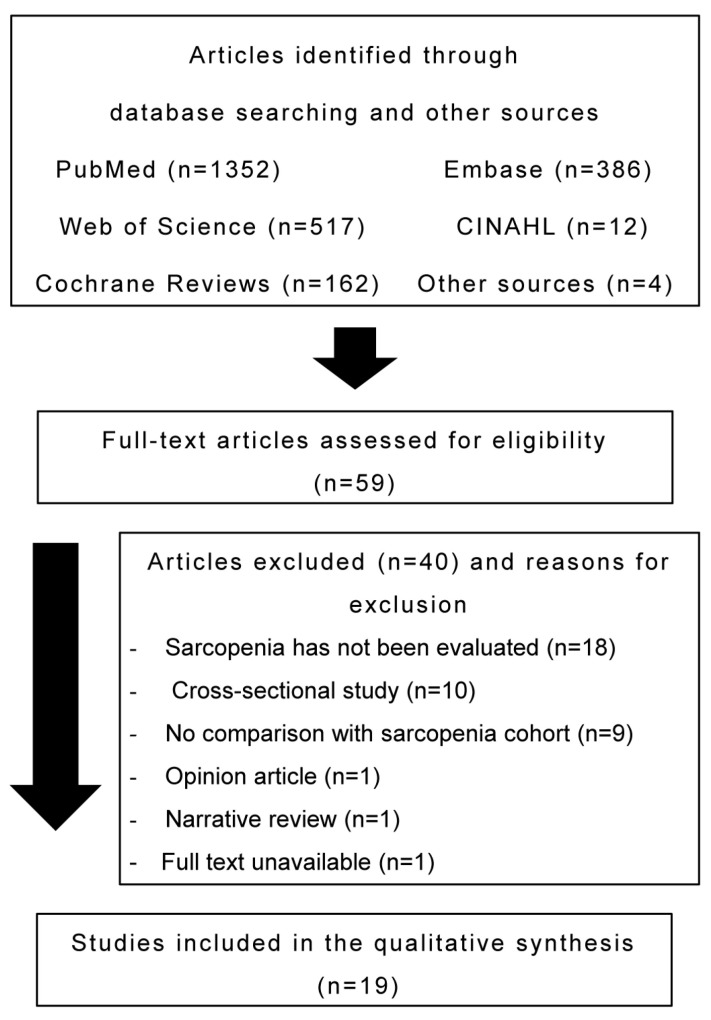
Flow diagram of the search process.

After screening the titles and abstracts of articles identified in the database search, 59 articles fulfilled the eligibility criteria. Of these, 40 articles were excluded as patients lacked a sarcopenia evaluation (n = 18), the study design was not appropriate (n = 12), no comparison was performed with the sarcopenia cohort (n = 9), or the full text was unavailable (n = 1). Ultimately, 19 articles were included in the present study. Figure 1 presents the flowchart of the literature search.

### 3.2. Characteristics of Included Studies

#### 3.2.1. Participant Characteristics

Most studies (n = 7 (36.8%) [10,11,12,13,14,15,16]) reported findings from Japan, while others reported findings from the United States (n = 5 [17,18,19,20,21]), Korea (n = 2 [22,23]), China (n = 2 [24,25]), Italy (n = 2 [26,27]), and Taiwan (n = 1 [8]). Table 2 summarizes the characteristics of the included articles. The target diseases were CSM (n = 2 [11,20]), LDSD (n = 9 [8,12,13,18,21,23,24,26,27]), lumbar spinal stenosis (LSS; n = 6 [10,14,15,16,22,25]), and ASD (n = 1 [19]). One study evaluated patients who underwent thoracolumbar surgery (n = 1 [17]).

#### 3.2.2. Study Characteristics

Regarding the publication language, excluding one report in Chinese [25], all the included articles were in English. Fifteen articles were retrospective observational studies [10,12,13,14,17,18,19,20,21,22,23,24,25,26,27], three were prospective observational studies [11,15,16], and one was a meta-analysis (LDSD) [8]. However, no intervention studies for SS and sarcopenia were available in any of the explored databases. 

#### 3.2.3. Sarcopenia Definition and Assessment Methods

All the identified studies assessed sarcopenia preoperatively. Regarding sarcopenia assessment in SS, five articles assessed muscle mass alone using dual-energy X-ray and bioelectrical impedance analysis [10,11,12,15,16]. The prevalence of sarcopenia, as defined by this assessment method, ranged between 20.3 and 47.4%.

Four articles evaluated the cross-sectional area of the psoas muscle [17,19,26,27]; the prevalence of sarcopenia ranged between 18.1 and 50.7%. Three articles evaluated sarcopenia according to the AWGS criteria [13,14,24], revealing that the prevalence of sarcopenia was 14.0, 20.0, and 23.1%, respectively. Two articles assessed sarcopenia based on the cross-sectional area of the erector spinae and psoas muscles, reporting a sarcopenia prevalence of 18.1 and 50.0%, respectively [18,25]. Another study defined sarcopenia by muscle quality, as assessed by magnetic resonance imaging (MRI), and reported a prevalence of 26.1% [20]. In addition, we included two articles that primarily examined physical function (grip strength) in patients with sarcopenia without assessing muscle mass, reporting prevalence rates of 38.2 and 50.0%, respectively [22,23]. One article examined sarcopenia using the International Statistical Classification of Diseases code and documented a sarcopenia prevalence of 4.5% [21]. 

#### 3.2.4. Study Outcomes 

Based on the studies included in the present review, the presence or absence of sarcopenia did not alter perioperative management. Five reports revealed that patients with SS and sarcopenia had decreased postoperative disease-specific QOL [10,12,20,22,24], whereas three reports found no significant postoperative differences between the sarcopenia and control groups [14,18,19]. A meta-analysis by Wu et al., which included several of the abovementioned studies, summarized the effects of sarcopenia on LDSD and found that the postoperative QOL was significantly lower in the sarcopenia group than that in the non-sarcopenia group [8]. 

Two articles reported a decrease in postoperative QOL in the sarcopenia group [11,22], whereas one detected no such difference [14]. One report documented poor improvement in postoperative pain changes in patients with LDSD in the sarcopenia group [25], and six found no differences in postoperative pain changes [10,12,13,14,18,24]. One report documented poor improvement in postoperative neck pain in patients with CSM in the sarcopenia group [20]. Additionally, two recorded a poor improvement in postoperative Japanese Orthopaedic Association (JOA) scores in the sarcopenia group [11,12], whereas one found no difference [13].

One article reported prolonged length of stay (LOS) after lumbar spine surgery in the sarcopenia group [23], and one found no such difference [19]. Two articles reported that the incidence of postoperative complications was significantly increased in the sarcopenia group after lumbar spine surgery [21,23], while three articles detected no difference [19,26,27]. Various other outcomes were evaluated after lumbar spine surgery, including postoperative costs [17], fall incidence [15], psychological factors [16], and motor function [22]; these outcomes were poorer in the sarcopenia group than in the non-sarcopenia group.

## 4. Discussion

Our literature review included 19 studies on sarcopenia and SS that examined postoperative outcomes as the main outcome measure. There were no articles on conservative management. Sarcopenia has been shown to negatively impact postoperative QOL in surgical patients. In addition, sarcopenia has a wide range of effects on surgical patients with SS, including increased risk of falls, deterioration of psychological factors, and impairment of postoperative physical function. Most studies were retrospective in design, and LDSD was the most frequently assessed disease. Imaging information derived from computed tomography (CT) and MRI is essential for the diagnosis and preoperative planning of LDSD; moreover, the associated muscle mass assessment is considered the gold standard [1]. LDSD was widely explored, and the number of publications on LDSD was the highest. This can be attributed to the ease of simultaneously performing disease-related and muscle mass assessments. Only two studies incorporated a sarcopenia cohort for CSM. One study included patients with ASD, while no study assessed patients with sarcopenia and scoliosis. At present, undertaking a systematic review of CSM, ASD, and scoliosis would be challenging owing to the limited number of reports. Additional studies on CSM, ASD, and scoliosis are needed to elucidate the relationship between SS and sarcopenia. 

Among the articles included in the current review, several reported worse disease-specific QOL in patients with CSM and LDSD in the sarcopenia cohort [10,12,20,22,24], which is consistent with the results of the previous meta-analysis [8]. Several included articles reported similar adverse outcomes, considering the occurrence of postoperative complications and falls in the sarcopenia group [15,21,23]. Sarcopenia has been found to increase adverse outcomes and decrease QOL owing to several factors, including muscle weakness and decreased immunity [28]. This phenomenon may occur in SS, which largely affects older individuals, suggesting that sarcopenia may negatively affect SS outcomes.

However, the current literature review was complicated by several challenges that made it difficult to define the impact of sarcopenia on SS. First, sarcopenia assessment was performed using diverse methods, including those employing imaging information such as CT or MRI [17,18,19,20,25,26,27], those that follow the AWGS criteria [12,13,14,15,16,24], and those that rely solely on grip strength as an indicator [22,23]. A meta-analysis has reported that the prevalence of sarcopenia varied between 10.0 and 27.0% in the general older population, depending on the diagnostic method used [29]. Likewise, the prevalence of sarcopenia varied widely among the studies included in the current review, ranging between 4.5 and 50.0%. The use of distinct methods to assess sarcopenia may lead to an overestimation or underestimation of sarcopenia prevalence.

Furthermore, the differences in assessment methods may influence the prevalence of sarcopenia in SS and its associated outcomes. Although most studies detected no difference in postoperative pain, i.e., the main symptom, between patients with and without sarcopenia [10,12,13,14,18,24], some reported worse postoperative pain in the sarcopenia group [20,25]. These inconsistent findings may be attributed to the different approaches used to assess muscle mass, focusing either on limb muscles or assessing the spinal muscles and their quality. In particular, spinal muscles are essential for controlling spinal movements and may directly impact spine-related symptoms [30]. Accordingly, whether the sarcopenia assessment is based on the limb or spinal muscle mass could have a substantial impact in the context of SS. Thus, the different methods used to assess sarcopenia may have contributed to discrepancies in certain outcomes, including postoperative outcomes and complications.

Second, the available literature on the association between SS and sarcopenia is predominantly from Asian countries, which raises the issue of racial bias. The prevalence of sarcopenia is estimated to be the highest among Asian populations across all diagnostic criteria, considering the global definition of sarcopenia [29]. This high prevalence among Asian populations may be influenced by the substantial interest of researchers in this region and the availability of diagnostic criteria specifically tailored for these populations, e.g., the AWGS criteria. It is predicted that the availability and use of region-specific diagnostic criteria, such as the AWGS, may have influenced these findings.

Importantly, the standardization of diagnostic criteria for sarcopenia internationally could lead to a reduction in racial bias and ensure a more accurate representation of the prevalence and impact of sarcopenia in different populations.

Finally, challenges related to the study design need to be addressed. Most articles included in the current review were retrospective studies, resulting in limited evidence based on the impact of sarcopenia on SS at this stage. However, two prospective studies showed negative effects [11,16], suggesting that a higher quality review could be conducted by prospectively investigating the impact of sarcopenia on SS. There have been no interventional trials specifically targeting sarcopenia in SS. One randomized controlled trial [31] evaluated a potential interventional strategy, revealing that a nutritional intervention focused on protein intake could improve postoperative trunk muscle mass in patients undergoing lumbar fusion surgery. Another study reported improvements in physical function following strength training or surgical intervention for lumbar disorders [14,32]. These findings suggest potential strategies to address sarcopenia in SS. In the future, interventional studies aimed at improving sarcopenia in SS should be conducted to assess their impact on important outcomes such as pain and disease-specific QOL.

The follow-up periods after spinal surgery varied between studies. To systematically elucidate the association between SS and sarcopenia, a preplanned prospective observational study should be conducted. In addition, a follow-up period of 2 years or longer should be considered to examine long-term outcomes, as a 2-year follow-up is a known criterion for postoperative outcomes after SS surgery [33].

Regarding the current assessment methods for sarcopenia in SS, this review showed that the assessment of spinal muscle morphology through imaging, which reflects the main symptoms of pain and postoperative QOL, is clinically useful. Therefore, evaluation using imaging information from CT or MRI is considered useful for clinical purposes. However, regarding the evaluation method, it is crucial to assess the muscle mass of both limb skeletal muscles and spinal muscles, determine which strongly influences clinical outcomes, and establish a clinically valuable evaluation method.

This study had several limitations. No meta-analysis or other quantification was performed owing to the number of outcomes and diseases included in this review and the variability in sarcopenia assessment. Additionally, no bias assessment was performed; therefore, selection or publication bias may be present. Therefore, the objectivity of the results cannot be guaranteed. Finally, the literature search explored limited databases, and we excluded populations from Latin America, Oceania, and Africa, generating a potential language selection bias.

## 5. Conclusions

Our literature review focused on studies exploring sarcopenia and SS and found that sarcopenia can negatively impact the QOL and postoperative outcomes in several diseases, including CSM and LDSD. However, there was no consensus among studies regarding the relationship between sarcopenia and the main symptoms of SS, such as pain. These discrepancies were attributed to the gap in internationally standardized sarcopenia assessment methods, potential racial bias, and variations in research design, which were identified as important limitations of the current study. The further accumulation of quality research is needed to clarify the relationship between SS and sarcopenia.

## Figures and Tables

**Table 1:** Search strategy for study selection.

#	Search Formula	Number of Hits
1	“Cervical spondylosis” OR “cervical myelopathy” OR “cervical spondylosis myelopathy” OR “cervical disc herniation” OR “cervical vertebrae” OR “cervical stenosis” OR “ossification of the posterior longitudinal ligament” OR “radiculopathy”	17,785
2	“Thoracic spine” OR “ankylosing spinal hyperostosis” OR “ossification of ligamentum flavum” OR “thoracic spondylotic myelopathy”	6770
3	“Lumbar spine” OR “lumbar spinal stenosis” OR “lumbar spinal canal stenosis” OR “lumbar spondylolisthesis” OR “spondylolisthesis” OR “lumbar degenerative disc disease” OR “degenerative lumbar spondylosis” OR “lumbar disc herniation”	45,851
4	“Adult spinal deformity” OR “scoliosis” OR “kyphoscoliosis” OR “kyphosis”	34,131
5	“Arthroscopy” OR “arthroplasty” OR “decompression” OR “fusion” OR “laminectomy” OR “laminoplasty” OR “lumbar surgery”	374,711
6	“Sarcopenia” OR “sarcopenic” OR “muscle mass” OR “muscle atrophy” OR “appendicular lean mass”	40,787
7	#1 OR #2 OR #3 OR #4 OR #5 AND #6	1352

The search strategy employed in PubMed is presented as an example.

**Table 2:** Summary of extracted studies.

Author, Year of Publication	Study Design	Population (Including Nationality, Disease, and Sex)	Definition and Assessment Methods of Sarcopenia	Subject Details	Outcomes	Key Findings of Results
Ko et al. (2022) [23]	Retrospective	Korea; patients who underwent elective posterior lumbar interbody fusion surgery (n = 225; male: female, 93:134)	HGS and SMI	Patients with low HGS (n = 86; mean age, 66.0 ± 9.0 years) Patients with normal HGS (n = 139; mean age, 65.0 ± 9.0 years) Prevalence of sarcopenia: 38.2%	LOS postoperative complications	A longer LOS (median 10 vs. 8 days) and a higher incidence of serious postoperative complications (15.1% vs. 3.6%) were observed in the low HGS group.
Kwon et al. (2020) [22]	Retrospective	Korea; patients who underwent spinal surgery for LSS (n = 278; male: female, 96:182)	HGS	Females in the low HGS group (n = 91; mean age, 69.5 ± 6.5 years); Males in the low HGS group (n = 48; mean age, 69.9 ± 6.5 years); Females in the high HGS group (n = 91; mean age, 64.5 ± 7.1 years); Males in the high HGS group (n = 48; mean age, 65.4 ± 9.7 years) Prevalence of sarcopenia: 50.0%	ODI, EQ-5D, VAS for back or leg pain, functional mobility tests (AST, SMT, TUG, STS)	In females, postoperative EQ-5D and ODI were clinically improved in the high HGS group.
Bokshan et al. (2017) [17]	Retrospective	Rhode Island (USA); patients who underwent thoracolumbar spine surgery (n = 50; male:female, 26:24)	Fell into the lowest tertile for their sex-specific TPA (the cross-sectional areas of the left and right psoas muscles at the level of the transverse process of L-4 on CT)	Patients with sarcopenia (n = 16; mean age, 76.6 ± 2.2 years) Patients without sarcopenia (n = 34; mean age, 70.8 ± 1.4 years) Prevalence of sarcopenia: 32.0%	Inpatient costs, transfusion rate, and rate of advanced imaging utilization	Patients with sarcopenia were 2.1 times as likely to require a blood transfusion (43.8% vs. 20.6%) and exhibited a 2.6-fold greater usage of advanced imaging (68.8% vs. 26.5%), associated with higher diagnostic imaging costs (USD 2452 vs. USD 801).
Inose et al. (2018) [12]	Retrospective	Japan; patients who underwent spinal surgery for LSS and lumbar compression fracture (n = 74; male:female, 33:52)	AWGS 2014 criteria SMI (DXA) determined by cutoff values only	Patients with sarcopenia (n = 37; mean age, 74.8 ± 0.9 years) Patients without sarcopenia (n = 37; mean age, 73.0 ± 1.0 years) Prevalence of sarcopenia: 47.4%	JOA score, VAS score (lower back pain, lower extremity pain, lower extremity numbness), patients who undergo rehabilitation	JOA scores (24.7 ± 0.4 vs. 23.0 ± 0.6) and recovery rates (68.6 ± 3.3 vs. 53.8 ± 5.2) at the final follow-up were significantly reduced in the sarcopenia subgroup.
Wu et al. (2021) [8]	Meta-analysis	Japan, Canada, the US, Korea; patients with LDSD (n = 1953) (Extracted from 14 studies)	None	None	Prevalence of sarcopenia, postoperative pain VAS, postoperative QOL	The overall prevalence of sarcopenia among patients with LDSD was 24.8% (95% CI, 17.3–34.3%). Patients with sarcopenia did not experience increased lower back and leg pain. However, lower QOL (SMD, −0.63; 95% CI, −0.84–−0.41) was observed postoperatively.
Koshimizu et al. (2018) [11]	Prospective	Japan; patients who underwent cervical laminoplasty (n = 171; male: female, 114: 57)	Sanada Classification SMI (DXA)	Patients with sarcopenia (n = 48; male:female, 37:11; mean age, 75.1 ± 8.9 years) Patients without sarcopenia (n = 123; male:female, 77:46; mean age, 70.3 ± 8.9 years) Prevalence of sarcopenia: 28.1%	JOA score, SF-36	The SF-36 score at 1 year postoperatively was higher in the non-sarcopenia group than that in the sarcopenia group. The JOA score was higher in the non-sarcopenia group at 1 year postoperatively.
McKenzie et al. (2019) [18]	Retrospective	USA; patients who underwent a single-level lumbar fusion for DS (n = 97; male:female, 46:51)	Sarcopenia is defined as 1 SD lower than the mean of the paraspinal muscle index by MRI	Patients with sarcopenia (n = 16; mean age, 64.6 ± 16.9 years) Patients without sarcopenia (n = 81; mean age, 61.6 ± 13.3 years) Prevalence of sarcopenia: 18.1%	ODI, SF-12 Physical (SF-12 P), SF-12 Mental (SF-12 M) and back pain VAS scores	No significant differences in ODI, SF-12, or back pain VAS scores.
Wada et al. (2020) [15]	Prospective	Japan; preoperative patients with LSS (n = 74; male:female, 36:38)	Low SMI (BIA) by AWGS 2019	Fallers (n = 24, median age, 73.0, range: 67.3–76.8 years) Non-fallers (n = 50; median age, 68.0, range: 63.0–76.0 years) Prevalence of sarcopenia: 20.3%	Falls occurred 12 months postoperatively	Preoperative low muscle mass predicted the occurrence of falls during the first 12 months after surgery (OR, 4.46; 95% CI, 1.02–19.63)
Wada et al. (2021) [16]	Prospective	Japan; preoperative patients with LSS (n = 73; male:female, 38:35)	Low SMI (BIA) by AWGS 2019	Patients with sarcopenia (n = 16; median age, 75.0; range, 70.3–81.3 years) Patients without sarcopenia (n = 57; median age, 68.0; range, 63.0–76.0 years) Prevalence of sarcopenia: 21.9%	NRS: leg pain, low back pain, JOA score, PCS, FABQ, HADS, walking velocity, HGS	The sarcopenia group had higher FABQ-PA scores than the normal group. Low muscle mass was significantly related to changes in the FABQ-PA score.
Akbik et al. (2022) [19]	Retrospective	USA; patients who underwent thoracolumbar ASD surgery (≥4 levels) (n = 235; male:female, 80:155)	The lowest quartile of PMI values measured at L3 by CT	Patients with sarcopenia (n = 59; mean age, 69.2 ± 9.6 years) Patients without sarcopenia (n = 176; mean age, 69.7 ± 7.4 years) Prevalence of sarcopenia: 25.1%	ODI, postoperative complications, LOS, reoperation, mortality	No significant differences in ODI, postoperative complications, LOS, reoperation, or mortality.
Eguchi et al. (2018) [10]	Retrospective	Japan; female patients who underwent surgery for LSS (n = 34)	Sanada Classification SMI (DXA)	Patients with Sarcopenia (n = 9) Patients with pre-sarcopenia (n = 12) Patients without sarcopenia (n = 13) Average age not stated Prevalence of sarcopenia: 26.5%	VAS for LBP, JOA score, RDQ	Patients with sarcopenia had lower RDQ at 6 months postoperatively than normal subjects.
Ruffilli et al. (2022) [26]	Retrospective	Italy; patients aged 50–85 years with LDSD treated with short posterior arthrodesis (3 levels or less) (n = 308; 148 male, 160 female)	PLVI by MRI (PLVI = (left psoas CSA + right psoas CSA)/2/L4 vertebral body CSA), which is the mean CSA of the psoas major divided by the mean area of the L4 vertebral body). PLVI is stratified into high and low groups at a cutoff value of 0.71 at baseline	Patients with sarcopenia (n = 153; mean age, 65.3 ± 6.4 years) Patients without sarcopenia (n = 155; mean age, 62.3 ± 5.7 years) Prevalence of sarcopenia: 49.7%	SSI	SSI was evenly distributed between low and high PLVI.
Sakai et al. (2020) [14]	Case-control study (retrospective)	Japan; patients with LSS who underwent surgical treatment (n = 235; male: female, 135:100)	AWGS2014 SMI by DXA	Patients with Sarcopenia (n = 33; mean age, 76.7 ± 5.9 years) Patients without sarcopenia (n = 171; mean age, 72.3 ± 5.5 years) Prevalence of sarcopenia: 14.0%	RDQ, EQ5D, SF36	Postoperatively, all three groups had good surgical outcomes. There were no significant differences between the sarcopenia and non-sarcopenia groups.
Barile et al. (2022) [27]	Retrospective	Italy; patients with LDSD who underwent a short (3 levels or less) posterior lumbar fusion (n = 304; male: female, 149:155)	PLVI by MRI (PLVI = (left psoas CSA + right psoas CSA)/2/L4 vertebral body CSA), which is the mean CSA of the psoas major divided by the mean area of the L4 vertebral body). PLVI is stratified into high and low groups at a cutoff value of 0.71 at baseline	Patients with sarcopenia (n = 154; mean age, 63.6 ± 5.9 years) Patients without sarcopenia (n = 150; mean age, 64.6 ± 6.0 years) Prevalence of sarcopenia: 50.7%	SSI	Sarcopenia (Low PLVI) was not associated with postoperative SSI
Pinter et al. (2022) [20]	Retrospective	USA; patients undergoing posterior cervical fusion from C2 to T2 for myelopathy with or without radiculopathy (n = 99; male:female, 55:44)	Goutalier classification of bilateral multifidus muscles at the C5-C6 level by MRI	Goutalier 0–1 (n = 28; mean age, 61.6 ± 9.0 years) Goutalier 1.5–2 (n = 5; mean age, 64.2 ± 9.2 years) Goutalier 2.5–4 (severe sarcopenia) (n = 26; mean age, 64.6 ± 8.9 years) Prevalence of sarcopenia: 26.1%	NDI, VAS neck scores, PROMIS Physical and Mental Component Scores	Patients with severe sarcopenia were more likely to report worsening of NDI, VAS Neck score, and PROMIS
Toyoda et al. (2019) [13]	Retrospective	Japan; patients aged >65 years who underwent minimally invasive lumbar decompression surgery (n = 130; male:female, 70:60)	SMI (BIA) by AWGS 2014	Patients with sarcopenia (n = 26; mean age, 80.9 ± 5.7 years) Dynapenia (n = 41; mean age, 77.5 ± 6.3) Patients without sarcopenia (n = 57; mean age, 74.6 ± 5.8 years) Prevalence of sarcopenia: 20.0%	JOA score VAS for leg pain, low back pain	No significant differences were observed between the sarcopenia and non-sarcopenia groups.
Li et al. (2021) [24]	Retrospective	China; patients who underwent a single-level stand-alone LLIF for lumbar diseases (n = 69; male:female, 28:41)	Low SMI (BIA) by AWGS 2019	Patients with sarcopenia (n = 16; median age, 66.5 years) Patients without sarcopenia subjects (n = 41; median age, 59.0 years) Prevalence of sarcopenia: 23.1%	ODI, back pain VAS	Postoperative ODI scores were higher in the sarcopenia group (35.1 vs. 25.1%) than those in normal subjects, and the percentage of ODI improvement was lower in the sarcopenia group (30.5% vs. 47.3%) than that in normal subjects. There was no significant difference in pain VAS.
Li et al. (2022) [25]	Retrospective	China; patients with LSS (n = 50; male: female, 22:28)	SMI at L3 level by CT (<45.4 cm^2^/m^2^ for males and <34.4 cm^2^/m^2^ for females) No description of the relevant muscle	Patients with sarcopenia (n = 25; male:female, 13:12; mean age, 64.8 years) Patients without sarcopenia (n = 25; male:female, 9:16; mean age 59.2 years) Prevalence of sarcopenia: 50.0%	Operative time, intraoperative blood loss, postoperative drainage volume, LOS, complications, pain VAS score, and ODI	The duration of hospitalization in the sarcopenia group was significantly longer than that in the non-sarcopenia group. Postoperative differences in VAS and ODI scores for lower back pain were significantly higher in the sarcopenia group than those in the non-sarcopenia group.
Albright et al. (2023) [21]	Retrospective	USA; patients who underwent index lumbar spine arthrodesis (n = 239,953; male: female, 104,319:135,634)	ICD code	Patients with sarcopenia (n = 1087; male:female, 519:568; mean age, 61.0 ± 13.7 years) Control patients (n = 1087; male:female, 530:557; mean age, 61.1 ± 13.2 years) Prevalence of sarcopenia: 4.5%	90-Day surgical and medical complication rates; cumulative rate of revision surgeries and readmission rates for all causes; and treatment costs	Patients with sarcopenia were more likely to be diagnosed with UTI (OR, 1.41) and to undergo incisional drainage (OR, 2.66). Patients with sarcopenia showed a high cumulative rehospitalization rate (OR, 1.24)

ASD, adult spinal deformity; AST, alternative step test; AWGS, Asian Working Group for Sarcopenia; BIA, bioelectrical impedance analysis; CI, confidence intervals; CSA, cross-sectional area; CT, computed tomography; DS, degenerative spondylolisthesis; DXA, dual-energy X-ray absorptiometry; EQ5D, EuroQol 5-dimensions; FABQ, fear avoidance brief questionnaire; HADS, hospital anxiety and depression scale; HGS, hand grip strength; ICD, International Statistical Classification of Diseases and Related Health Problems; JOA, Japanese Orthopaedic Association; LDSD, lumbar degenerative spine disease; LLIF; lateral lumbar interbody fusion; LOS, length of stay; LSS, lumbar spinal stenosis; MRI, magnetic resonance imaging; NDI, neck disability index; ODI, Oswestry Disability Index; OR, odds ratio; PCS, pain catastrophizing scale; PLVI, psoas to L4 vertebral body index; PMI, psoas muscle index; PROMIS, patient-reported outcome measurement information system; QOL, quality of life; RDQ, Roland–Morris Disability Questionnaire; SD, standard deviation; SF-36, short-form 36; SMD, standardized mean difference; SMI, skeletal muscle mass index; SMT, six-meter walk test; SSI, surgical site infection; STS, sit to stand test; TPA, total psoas muscle area; TUG, timed up and go test; USA, United States of America; UTI, urinary tract infection; VAS, visual analogue scale.

## Data Availability

The data presented in this study are available on request from the corresponding author. Public data sharing is not applicable to this article due to ethical considerations and privacy restrictions.
